# Effect of Mechanical Heterogeneity on Strain and Stress Fields at Crack Tips of SCC in Dissimilar Metal Welded Joints

**DOI:** 10.3390/ma14164450

**Published:** 2021-08-09

**Authors:** Shun Zhang, He Xue, Shuai Wang, Yuman Sun, Fuqiang Yang, Yubiao Zhang

**Affiliations:** 1 School of Mechanical Engineering, Xi’an University of Science and Technology, Xi’an 710054, China; 19105016005@stu.xust.edu.cn (S.Z.); 17101016005@stu.xust.edu.cn (S.W.); 19205201061@stu.xust.edu.cn (Y.S.); 15109212717@163.com (Y.Z.); 2 School of Science, Xi’an University of Science and Technology, Xi’an 710054, China; yang_afreet@163.com

**Keywords:** strength mismatch, work hardening mismatch, stress corrosion cracking, strain and stress fields, synergistic effect

## Abstract

The crack tip strain and stress condition are one of the main factors affecting stress corrosion cracking (SCC) behaviors in the dissimilar metal welded joint of the primary circuit in the pressurized water reactor. The mechanical property mismatch of base metal and weld metal can significantly affect the stress and strain condition around the crack tip. To understand the effect of different weld metals on strain and stress fields at SCC crack tips, the effects of strength mismatch, work hardening mismatch, and their synergy on the strain and stress field of SCC in the bi-material interface, including plastic zone, stress state, and corresponding *J*-integral, are investigated in small-scale yielding using the finite element method. The results show a significant effect of the strength mismatch and work hardening mismatch on the plastic zone and stress state in the weld metal and a negligible effect in the base metal. *J*-integral decreases with the single increase in either strength mismatch or work hardening mismatch. Either the increase in strength mismatch or work hardening mismatch will inhibit the other’s effect on the *J*-integral, and a synthetic mismatch factor can express this synergistic effect.

## 1. Introduction

As the operating time of nuclear power plants increases, stress corrosion cracking (SCC) has become a widespread issue during the last few decades [1]. SCC is a slow crack growth process under the combination of environment, material, and load stress in the crack tip area [2]. Because of the heterogeneous microstructure and mechanical properties in the bi-material interface regions, the dissimilar metal weld joint (DMWJ) is more susceptible to SCC and becomes the weakest link of structural materials in the pressurized water reactor (PWR) [3,4]. The stress and strain condition around the crack tip is a critical factor affecting the SCC behaviors [1]. Therefore, it is essential to calculate strain and stress fields at the crack tip of SCC for predicting the crack growth direction and rate of SCC quantitatively. The mechanical property mismatch is an important factor to be considered because it can significantly affect the stress and strain condition around the crack tip.

In general, the mechanical property mismatch is defined by the strength mismatch ratio of the yield strength of the weld metal and base metal. Many efforts were made regarding the effect of strength mismatch on the crack-tip stress and strain field [5] and corresponding crack driving force [6,7], crack-tip constraint [8,9]. In most of these studies, the default weld metal and base metal have the same hardening properties, i.e., the true stress–strain curves of two metals after yielding are parallel. Nevertheless, a few studies showed that the work hardening mismatch could significantly affect the crack-tip stress and strain fields and crack driving force with the same yield strength [10,11,12]. In fact, the yield strength and work hardening of the base metal and weld metal are mismatched simultaneously in the DMWJ. Due to the use of different base metals and weld metals, the mismatch states are different, and the degradation of materials in the welded joint will occur and change the macro mechanical properties due to the influence of extreme environments such as irradiation and high temperature [13]. Moreover, the “local mismatch” was proposed meaning the difference of yield strength and work hardening near the crack [14,15]. Therefore, the effect of mechanical property mismatch on the crack-tip stress and strain field of SCC needs to be studied in more detail.

However, when it comes to the effects of strength mismatch and work hardening mismatch on the crack-tip stress and strain field and crack driving force, it can be found that previous studies focused on one of them with the other unchanged or ignored. The synergy (inhibition or promotion) of these two mismatches has not been studied. In the present study, the effects of strength mismatch, work hardening mismatch, and their synergy on the strain and stress field of interface SCC crack tip are investigated in small-scale yielding by the finite element method (FEM) based on elastic–plastic fracture mechanics (EPFM) approach. The plastic zone and stress level near the crack tip and corresponding *J*-integral are also discussed. The results presented in this work can provide more detailed recommendations for selecting welding materials and evaluating the structural integrity of the DMWJ from the perspective of the SCC crack driving force.

## 2. Materials and Methods

### 2.1. Materials and Specimen Geometry

The Ramberg-Osgood equation can be used to express the true stress–strain (*σ*-*ε*) curves of both the weld metal and base metal in the DMWJ, which is written as

(1) $$\frac{\varepsilon }{\varepsilon_{0}}=\frac{\sigma }{\sigma_{0}}+\alpha {\left(\frac{\sigma }{\sigma_{0}}\right)}^{n},$$

where *σ*_0_ is the yield strength, *ε*_0_ is the yield strain, *α* is the Ramberg–Osgood coefficient, and *n* is the work hardening exponent. The safe-end DMWJ is utilized to join the ferric steel pipe-nozzle of the pressure vessel with the austenitic stainless steel safe-end. Ferritic low alloy steel A508, usually used for the pipe-nozzle of the nuclear pressure vessel, is selected as the base metal. The material mechanical parameters of A508 are Young’s modulus *E* = 183,000 MPa, Poisson’s ratio *ν* = 0.3, yield stress *σ*_0_ = 410 MPa, strain hardening exponent *n* = 5.41, and the Ramberg–Osgood coefficient *α* = 5.11 at the operating temperature of about 340 °C [16,17]. At present, the world’s uses of safe-end weld metal are 309L/308L, Inconel 82, and Inconel 152 in the United States; Soudonel 690 in Belgium; Thermanit 690 in Germany; Nic 703D in Japan; and Sanicro 71 in Sweden. They all have different yield strengths and work hardening coefficients. For example, the yield strength of alloy 82 is 315 MPa, and the work hardening coefficient is 7.01 at 320 °C [18]; the yield strength of alloy 52 is 380 MPa, and the work hardening coefficient is 3.29 at 340 °C [19]; the yield strength of 309L/308L is 270 MPa, and the work hardening coefficient is 3.74 at 295 °C [20]. Therefore, a wide range of the strength and work hardening mismatch ratio should be selected in this study for almost covering the yield strength and work hardening coefficient of all weld metal.

To investigate effects of strength mismatch, work hardening mismatch, and their synergy on the strain and stress field, it is assumed that Young’s modulus *E*, Poisson’s ratio *ν*, and the Ramberg–Osgood coefficient *α* in the weld metal are consistent with base metal, but the yield strength and work hardening exponent vary in the weld metal. The strength mismatch ratio is usually defined as

(2) $$M_{S}=\frac{\sigma_{W}}{\sigma_{B}},$$

where *σ_W_* and *σ_B_* are the yielding strength of the weld metal and base metal. *M_S_* varies from 0.7 to 1.3, which is considered to be a typical range [21]. The *M_S_* is 0.7, 0.85, 1, 1.15, and 1.3, and the corresponding yield strength of the weld metal is 287, 348.5, 410, 471, and 533 MPa in this study. Similar to the strength mismatch, the work hardening mismatch ratio is defined as

(3) $$M_{N}=\frac{n_{W}}{n_{B}},$$

where *n_W_* and *n_B_* are the work hardening exponent of the weld metal and base metal, respectively. The range of *M_N_* from 0.5 to 1.8 is considered, and the value interval is 0.1. Therefore, the work hardening exponent of the weld metal varies from 2.71 to 9.74, which is approximately coincident with the variation range of work hardening exponent in other studies [22,23].

**Figure 1:**
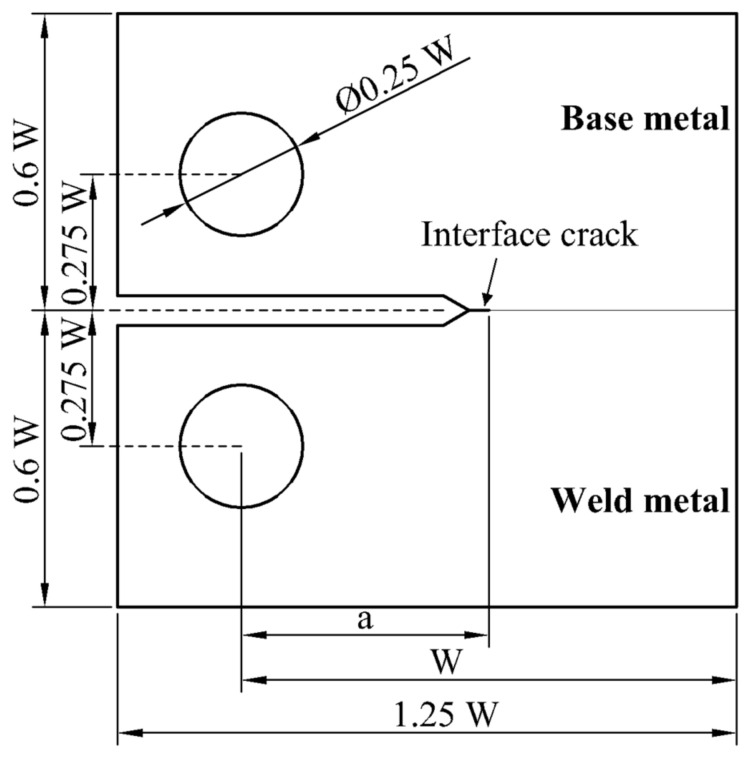
Geometry of 1T-CT specimen.

As shown in Figure 1, according to ASTM E399-17 [24], 1T-CT welded joint specimen (W = 50 mm, a = 0.5 W) was used in finite element analysis, which is often used in SCC experiments [25]. The crack is located at the interface of the weld metal and base metal.

### 2.2. FEM Model

**Figure 2:**
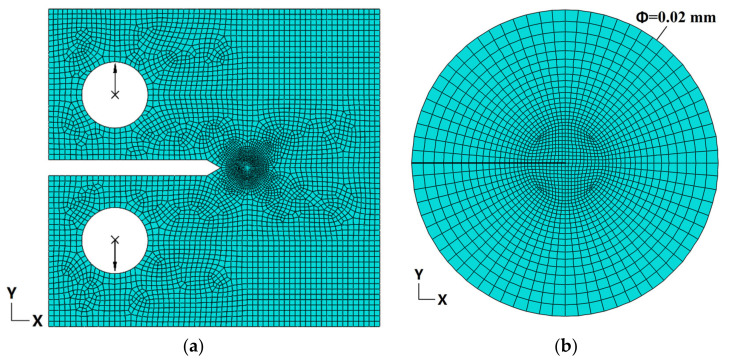
Finite element model of CT specimen. (**a**) The whole model and (**b**) meshes around the crack tip.

The two-dimensional finite element model with 10738 8-node biquadrate plane strain quadrilateral (CPE8) elements is shown in Figure 2. The meshes around the crack tip were refined with 1728 CPE8 elements, as shown in Figure 2b. The crack growth direction is the X direction in the coordinate system. The finite element model was calculated by ABAQUS code using the incremental theory of plasticity. The stress intensity factor (*K*) is usually used as a parameter to measure the external load of the CT specimen in SCC experiments. To investigate the synergistic effect of two mismatches on the strain and stress field under the same load, *K* is set as constant 30 MPa·m^1/2,^ which is usually used in SCC experiments [26,27]. The specimen was loaded by concentrated force in the center point of two loaded holes in the vertical direction, and all other motions of the center point were restrained except the vertical direction. The coupling constraint was defined between the center point and the loaded hole.

## 3. Results and Discussion

### 3.1. Plastic Zone

**Figure 3:**
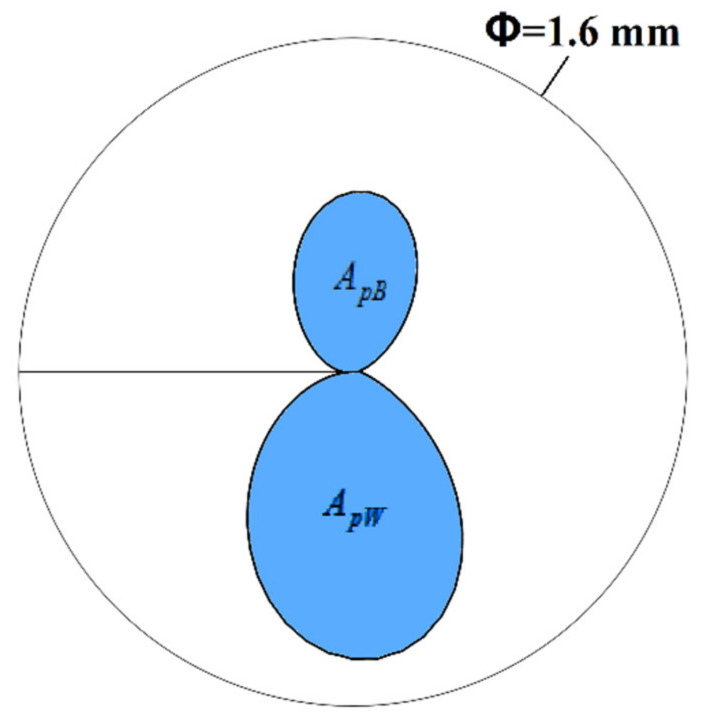
Diagram of plastic zone area of the base metal and weld metal.

The local stress and strain distribution near the crack tip can significantly affect the SCC growth rate and direction. Therefore, they are usually used as mechanical parameters to predict the SCC behavior quantitatively [28,29]. The plastic zone around the crack tip is investigated in this section, and the stress distribution will be investigated next section. To investigate the plastic zone on both sides of the SCC crack, as shown in Figure 3, the plastic zone area (surrounded by 0.2% equivalent plastic strain isoline) of the weld metal and base metal are defined and expressed by *A_pW_* and *A_pB_*, respectively.

**Figure 4:**
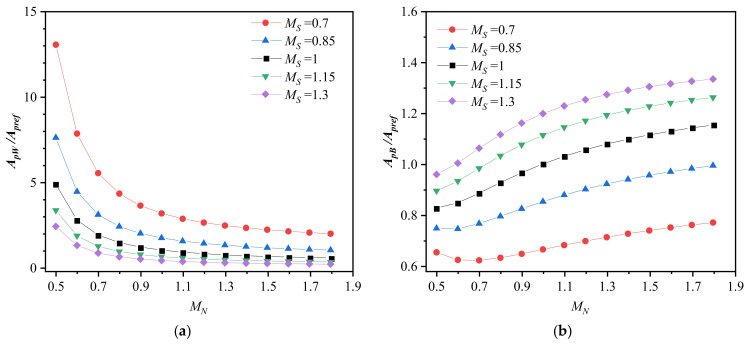
Plastic zone area. (**a**) Weld metal and (**b**) base metal.

The plastic zone area of the weld metal and base metal are shown in Figure 4, where *A_pW_* and *A_pB_* are normalized by the plastic zone area of any side homogeneous base metal (*M_S_* = *M_N_* = 1) *A_pref_*. It can be seen from Figure 4a that when work hardening is evenly matched (*M_N_* = 1), the plastic zone area of the weld metal decreases with increasing strength mismatch ratio, consisting with the study of Lee and Kim [30]. When yield strength is evenly matched (*M_S_* = 1), the plastic zone area of the weld metal also decreases with increasing work hardening mismatch ratio. Meanwhile, with increasing *M_S_* from 0.7 to 1.3, the change degree of *A_pW_* with *M_N_* is reduced. Additionally, the change degree of *A_pW_* with *M_S_* is reduced with increasing *M_N_* from 0.5 to 1.8. It indicates that when the yield strength and work hardening exponent are mismatched simultaneously, the increase in either inhibits the effect of the other on the plastic zone area of the weld metal. Figure 4b shows that when work hardening is evenly matched (*M_N_* = 1), the plastic zone area of the base metal increases with increasing *M_S_*. When yield strength is evenly matched (*M_S_* = 1), the plastic zone area of the base metal also increases with increasing *M_N_*, which is opposite to the trend of change in the weld metal. This opposite phenomenon can be explained as the increase in the plastic zone in the weld metal releases more high stress at the crack tip. Therefore, the high stress to be released in the base metal decreases, the plastic zone of the base metal decreases correspondingly, and vice versa. Those results are consistent with the other studies [29,30]. However, when comparing Figure 4b with Figure 4a, the variation in the plastic zone area in the base metal (from 0.62 to 1.33) is much smaller than weld metal (from 0.17 to 13.06). Thus the effects of strength mismatch and work hardening mismatch on the plastic zone area are negligible in the base metal but significant in the weld metal.

**Figure 5:**
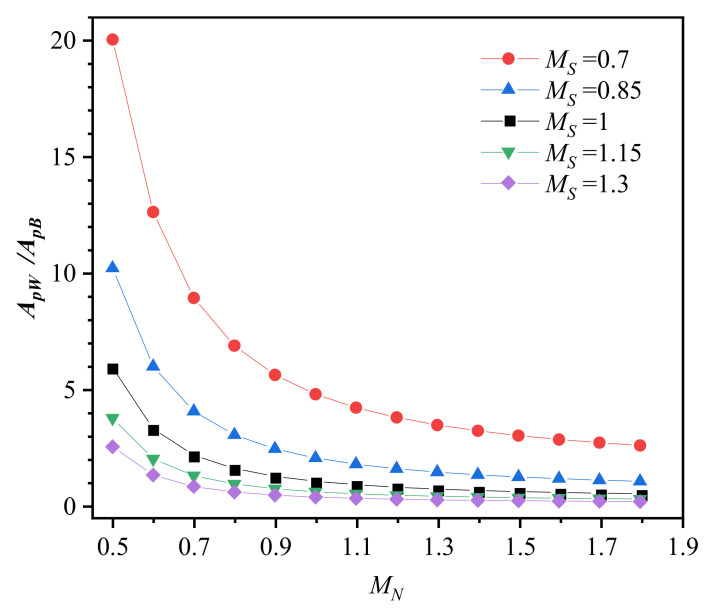
The ratio between plastic zone area of the weld metal and base metal.

It is assumed that the plastic zone area is regarded as the crack driving force in addition to the applied loading [15]. Therefore, the ratio of *A_pW_* and *A_pB_* is greater than 1, indicating that the crack driving force in the weld metal is greater than the base metal, and the crack might grow into the weld metal and vice versa. Figure 5 shows the ratio between the plastic zone area of the weld metal and base metal. The ratio of *A_pW_* and *A_pB_* is dominated by the *A_pW_* as the variation in *A_pW_* is much greater than *A_pB_*. The results in Figure 5 further indicate that the crack growth direction depends on both strength mismatch and work hardening mismatch. For example, the crack might grow into the base metal when *M_S_* > 1, without considering the work hardening mismatch (*M_N_* = 1), but into the weld metal when *M_N_* = 0.7. Therefore, it is not enough to consider one mismatch only for estimating the crack growth direction, and the synergistic effect of strength mismatch and work hardening mismatch must be considered.

### 3.2. Stress Distribution

For convenience, the “point match” method, i.e., selecting one or several points near the crack tip to characterize the stress level [31], is used to study the stress field at the crack tip. Additionally, the 20 μm can be used as the distance from the characteristic point to the crack tip of the SCC [2]. Therefore, to investigate stress level on both sides of the SCC crack, two points in *r* = 0.02 mm, *θ* = ±90°, are selected in this study, where *r* and *θ* are the polar coordinates centered at the crack tip with *θ* = 0° corresponding to the interface. The stress level in the base metal and weld metal is represented by the Von Mises stress at these two points and is expressed by *σ_VB_* and *σ_VW_*.

**Figure 6:**
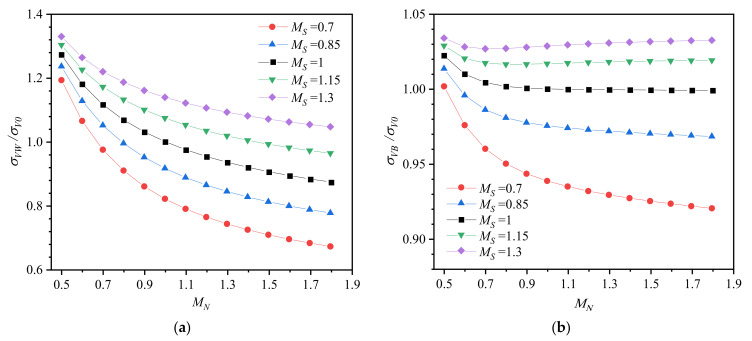
Von Mises stress. (**a**) Weld metal and (**b**) base metal.

Figure 6 shows the Von Mises stress in the weld metal and base metal for different strength mismatches and work hardening mismatches, where *σ_VB_* and *σ_VW_* are normalized by the Von Mises stress of the homogeneous base metal (*M_S_* = *M_N_* = 1) *σ_V0_* in *r*= 0.02 mm, *θ* = 90°. The results in Figure 6a suggest that when work hardening is evenly matched (*M_N_* = 1), the Von Mises stress in the weld metal increases with increasing strength mismatch ratio *M_S_*, consistent with other studies [5,29]. When yield strength is evenly matched (*M_S_* = 1), the Von Mises stress in the weld metal decreases with increasing work hardening mismatch ratio *M_N_*. Meanwhile, the change degree of *σ_VW_* with *M_N_* decreases with increasing *M_S_* (from 0.7 to 1.3), and the change degree of *σ_VW_* with *M_S_* increases with increasing *M_N_* (from 0.5 to 1.8). It indicates that when the yield strength and work hardening exponent are mismatched simultaneously, the increase in *M_S_* inhibits the effect of *M_N_* on the stress level in the weld metal. The increase in *M_N_* promotes the effect of *M_S_* on the stress level in the weld metal. Figure 6b shows that the tread of *σ_VB_* is similar to that of *σ_VW_*, which is because the stresses in the weld metal and base metal are similar to the relationship between force and reaction force. However, the maximum variation in *σ_VB_*, which occurs in *M_S_* = 0.7 and *M_N_* = 1.8, is less than 8% compared with the homogeneous base metal. It implies that the effects of strength mismatch and work hardening mismatch on stress in the base metal can be neglected compared with variation in stress level in the weld metal (33%).

**Figure 7:**
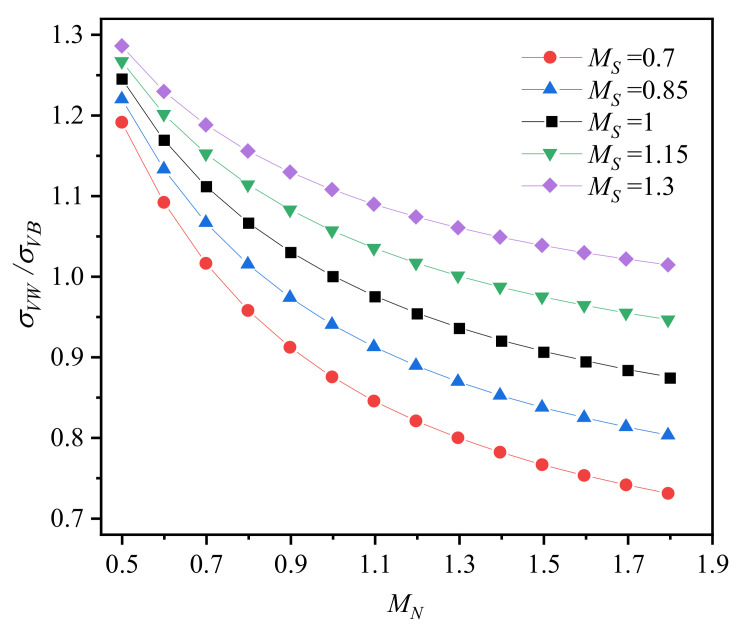
The ratio between Von Mises stress in the weld metal and base metal.

To compare the Von Mises stress in the weld metal and base metal, Figure 7 illustrates the ratio between them. The variation in *σ_VW_*/*σ_VB_* is consistent with the variation in *σ_VW_* (Figure 6a) because the ratio of *σ_VB_* and *σ_VW_* is dominated by *σ_VW_*. As *M_S_* changes, the *σ_VW_*/*σ_VB_* curve is not a simple translation. It demonstrates that the synergistic effect of strength mismatch and work hardening mismatch on stress distribution at the crack tip is significant.

### 3.3. J-Integral

The stress and strain fields at the crack tip are not in accordance with the HRR (Hutchinson–Rice–Rosengren) field distribution due to the mechanical property mismatch of the base metal and weld metal. However, it is notable that the *J*-integral is still an effective parameter to measure the scale of stress and strain region at the SCC crack tip [32]. When the stress intensity factor (*K*) is constant, the mechanical property mismatch affects the *J*-integral.

**Figure 8:**
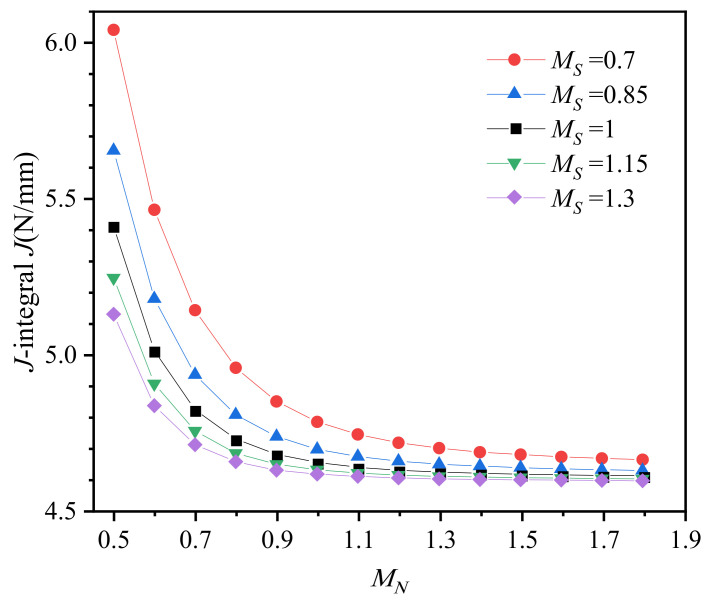
*J*-integral with different strength and strain hardening mismatches.

Figure 8 shows the *J*-integral with different strength mismatches and work hardening mismatches. The *J*-integral for homogeneous base metal (*M_S_* = *M_N_* = 1) is 4.651, and its value is 4.602, calculated by the EPRI (Electric Power Research Institute) approach. The relative error is 1.07%, which demonstrates that the FEM analyses are reliable in this study. The *J*-integral decreases with increasing strength mismatch ratio *M_S_* when work hardening is evenly matched (*M_N_* = 1), which is consistent with Lee’s research [6]. When yield strength is evenly matched (*M_S_* = 1), the *J*-integral also decreases with increasing work hardening mismatch ratio *M_N_*, and the variation range less than 1% when *M_N_* > 1. In addition, with increasing *M_S_* from 0.7 to 1.3, the *J*-integral curve to *M_N_* does not simply translate, and the change in *J*-integral reduces from 29.6% to 11.7%. With increasing *M_N_* from 0.5 to 1.8, the change in *J*-integral with *M_S_* reduces from 17.8% to 1.5%. Therefore, when the yield strength and work hardening exponent are mismatched simultaneously, the increase in either will inhibit the effect of the other on *J*-integral rather than superimposed on each other simply. The results in Figure 8 further indicate that higher yield strength and work hardening coefficient of weld metal will lead to a smaller crack driving force (*J*-integral) for interface SCC crack.

Further, in order to characterize the synergistic effect of strength mismatch and work hardening mismatch on *J*-integral, the mismatch factor is attempted to define as

(4) $$M_{2}=M_{N}\sqrt{M_{S}},$$

**Figure 9:**
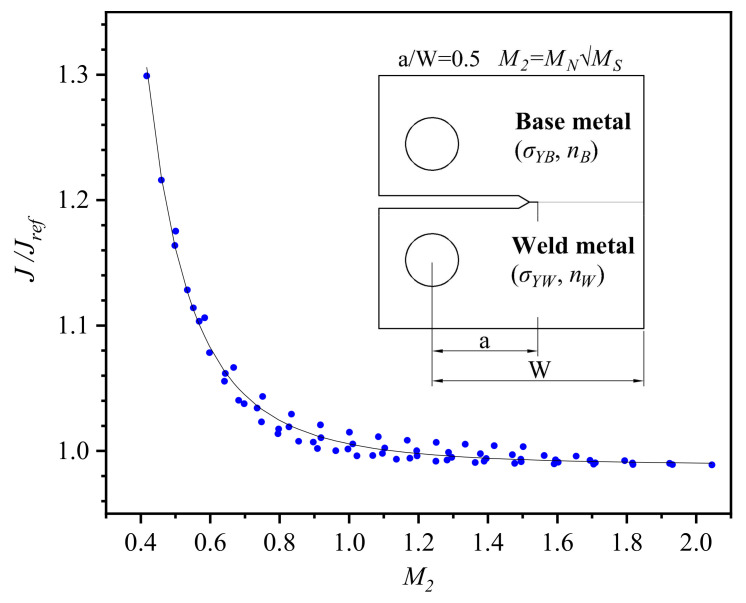
Fitting function of *J*-integral with different mismatch factors.

Figure 9 shows a fitting empirical equation curve of *J*-integral about *M*_2_ based on Figure 8, where the *J*-integral is normalized by the *J*-integral of homogeneous base metal *J_ref_* for engineering applications. There is an excellent fitting effect between *J*/*J_ref_* and *M*_2_, and the equation is expressed as

(5) $$J/J_{ref}=aM_{2}^{b}+c,$$

where *a* = 0.01709, *b* = −3.358, and *c* = 0.9872, the coefficient of correlation *R*-square (*R*^2^) is 0.988. Figure 9 further indicates that the *J*-integral decreases as a power function as the mismatch factor *M*_2_ increases, and the variation (less than 1.6%) in *J*-integral can be neglected when *M*_2_ > 1.

**Figure 10:**
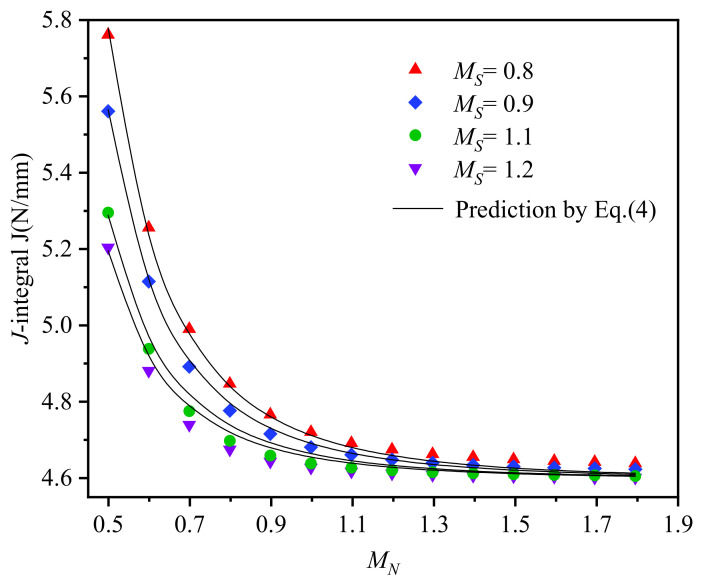
Comparison between the FEA results and predicted *J*-integral.

Figure 10 illustrates the comparison between the FEA results and predicted *J*-integral values with *M_S_* = 0.8, 0.9, 1.1, and 1.2. Good agreements are achieved, and the maximum relative error is less than 1%. Therefore, *M*_2_ quantifies the interdependencies of effects among strength mismatch and work hardening mismatch on *J*-integral and might be a suitable strategy to estimate the *J*-integral of interface SCC crack for DMWJs. For example, after *J*-integral of homogeneous base metal *J_ref_* is obtained by the Engineering Treatment Model (ETM) or EPRI, the *J*-integral of interface crack for DMWJs can be estimated according to the mismatch factor *M*_2_ between the weld metal and base metal. However, it should be noted that as a practical attempt to characterize the synergistic effect of strength mismatch and work hardening mismatch on *J*-integral, *M*_2_ and corresponding Equation (5) are applicable in CT specimen with small scale yielding in this research. The effects of constraints by different geometries and loads on *M*_2_ need to be further investigated.

The *J*-integral, with respect to strength mismatch application, was extended based on the EPRI approach [8,33] and used in the structural integrity assessment, such as R6 or BS 7910. However, this study demonstrates that the synergistic effect of strength mismatch and work hardening mismatch on the *J*-integral is significant and must be considered in small-scale yielding. To consider only one effect would result in a conservative or non-conservative result. Therefore, it is recommended to calculate the crack driving force of SCC related to both strength and hardening mismatch in the integrity design and evaluation of the DMWJ in nuclear power plants.

## 4. Conclusions

In conclusion, the synergistic effect of strength mismatch and work hardening mismatch on the strain and stress fields of SCC crack tip, including plastic zone, stress state, and corresponding *J*-integral, were investigated in small-scale yielding by FEM numerical analyses based on the EPFM approach. The following main conclusions were drawn from the results obtained:

- The effect of strength mismatch and work hardening mismatch on the plastic zone area is negligible in the base metal but significant in the weld metal. The increase in either will inhibit the effect of the other on the plastic zone area of the weld metal. The crack growth direction depends on the synergistic effect of two mismatches;
- The effect of strength mismatch and work hardening mismatch on the stress distribution is negligible in the base metal but significant in the weld metal. The increase in strength mismatch ratio will inhibit the effect of the work hardening mismatch ratio on the stress level of the weld metal. The increase in work hardening mismatch ratio will promote the effect of the strength mismatch ratio on the stress level of the weld metal;
- *J*-integral decreases with the single increase in either strength mismatch or work hardening mismatch. Either the increase in strength mismatch or work hardening mismatch will inhibit the other’s effect on the *J*-integral, and this synergistic effect may be expressed by a synthetic mismatch factor *M*_2_. Further study of the mismatch factor *M*_2_ and its use are currently being carried out;
- Higher yield strength and work hardening coefficient of weld metal will reduce the crack driving force (including plastic zone and *J*-integral) for interface SCC crack. It is recommended to calculate the strain and stress field and crack driving force of SCC for both strength and hardening mismatch in structural integrity assessment.

## Data Availability

The data presented in this study are available on request from the corresponding author.
